# Therapeutic potential of combining mirogabalin with antidepressants in relieving hypersensitivity: evidence from a mouse model of neuropathic pain

**DOI:** 10.1007/s43440-026-00862-6

**Published:** 2026-05-28

**Authors:** Renata Zajączkowska, Agata Ciechanowska, Katarzyna Pawlik, Katarzyna Ciapała, Wioletta Makuch, Jerzy Wordliczek, Magdalena Kocot-Kępska, Joanna Mika

**Affiliations:** 1 Department of Interdisciplinary Intensive Care, Jagiellonian University Medical College, św. Anny 12, Kraków, 31-008 Poland; 2 Department of Pain Pharmacology, Maj Institute of Pharmacology, Polish Academy of Sciences, Smętna 12, Kraków, 31-343 Poland; 3 Department of Pain Research and Treatment, Jagiellonian University Medical College, św. Anny 12, Kraków, 31-008 Poland

**Keywords:** Mirogabalin, Amitriptyline, Duloxetine, Neuropathic pain, Mice, Anti-nociception

## Abstract

**Background:**

Neuropathic pain remains a significant clinical problem that necessitates the development of more effective treatment options. Compared with commonly used clinical drugs, such as gabapentin and pregabalin, mirogabalin is a novel gabapentinoid that exhibits greater affinity and selectivity for the α2δ-1/-2 subunits of voltage-gated calcium channels, properties associated with strong nociceptive modulation. The aim of this study was to determine whether and how mirogabalin influences neuropathy symptoms and the antinociceptive effects of antidepressants (amitriptyline and duloxetine).

**Methods:**

Studies were conducted in mice after chronic constriction injury of the sciatic nerve. On Day 7, a single intraperitoneal administration of mirogabalin (5–60 mg/kg), amitriptyline (1–10 mg/kg), or duloxetine (10–30 mg/kg) at various doses was performed. Additionally, mirogabalin (10 mg/kg) was chronically injected twice daily, while amitriptyline (5 mg/kg) or duloxetine (10 mg/kg) was administered once daily. Hypersensitivity was assessed using the von Frey and the cold plate tests.

**Results:**

Using a mouse model of neuropathy, a single injection of mirogabalin, amitriptyline, or duloxetine reduced tactile and thermal hypersensitivity to a similar degree, with the most durable effect observed with mirogabalin, persisting up to 6 hours. Repeated twice-daily intraperitoneal administration of mirogabalin combined with once-daily intraperitoneal dosing of amitriptyline or duloxetine resulted in a potentiated anti-nociceptive response in the used model.

**Conclusions:**

These results may serve as a basis for further evaluation of the potential use of mirogabalin in the clinical treatment of neuropathic pain. Furthermore, they suggest that using mirogabalin together with amitriptyline or duloxetine may provide significant therapeutic benefits, enabling the use of lower doses of these medications, which may reduce the risk of adverse events.

**Graphical Abstract:**

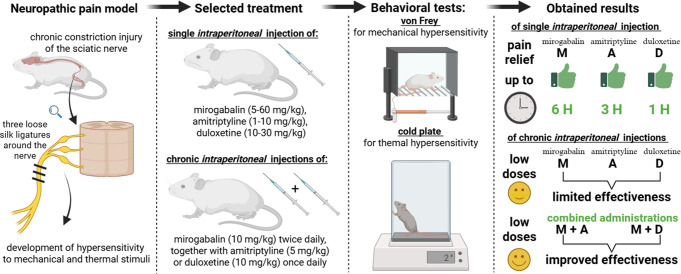

## Highlights

**In a mouse model of neuropathy evoked by chronic constriction of the sciatic nerve**

**After a single intraperitoneal administration, mirogabalin provides the following**

•A good anti-nociceptive effect, similar to that of amitriptyline and duloxetine

•A longer-lasting anti-nociceptive effect (up to 6h) compared with amitriptyline/duloxetine

**The combination of low doses of mirogabalin with amitriptyline/duloxetine resulted in improved anti-nociceptive action**

## Introduction

Neuropathic pain remains a very serious clinical problem, affecting up to 7–10% of the population, impacting not only the physical but also the mental health of patients [1–3]. This type of pain results from both damage to and diseases of the sensory–somatic nervous system [1, 4]. First-line therapies include gabapentinoids (e.g., gabapentin, pregabalin), which target voltage-gated calcium channels (VGCCs), tricyclic antidepressants (TCAs; e.g., amitriptyline), and serotonin-norepinephrine reuptake inhibitors (SNRIs; e.g., duloxetine) [3, 5, 6]. Notably, clinical data indicate that neuropathic pain treatment is effective in only 30–50% of patients [7]. Therefore, experimental research is being conducted to identify innovative drugs and effective combinations of new and old drugs that could be used clinically to improve this unfavorable situation.

One such new drug recently introduced in some Asian countries into clinical practice is mirogabalin [8–13], a gabapentinoid that, compared with clinically used drugs (gabapentin/pregabalin), binds with greater strength and selectivity to the α2δ-1/α2δ-2 subunits of VGCCs [14]. Data from the literature indicate that mirogabalin has better anti-nociceptive properties and reduces the incidence of adverse effects compared with gabapentin and pregabalin [15]. In 2019, mirogabalin was approved in Japan for the treatment of postherpetic neuralgia and diabetic neuropathy [8]. The prospective, multi-center study conducted in Japan in 2021 demonstrated that switching from pregabalin to mirogabalin is well tolerated and effective for the treatment of neuropathic pain [9].

The second key group of drugs used as first-line treatments are TCAs and SNRIs, which diminish nociceptive transmission by inhibiting serotonin and norepinephrine reuptake [3, 6]. Amitriptyline, a medication from the TCA group, is frequently used to treat neuropathic pain [6]. For patients who may be intolerant to TCAs, SNRIs such as duloxetine are alternatives that are typically chosen because of their favorable side effect profile compared with those of other drugs [6]. The choice among gabapentinoids, TCAs, and SNRIs requires an individualized approach, considering patient-specific factors such as comorbidities, age, pain characteristics, and failures or successes of the prior treatment [3, 5, 6]. Importantly, when monotherapy does not provide sufficient pain relief, combinations of gabapentinoids with TCAs or SNRIs are often used [3, 5, 6, 16–19]. To date, experimental data on the effects of combining mirogabalin with other medications are very sparse. Our previous study conducted in rodents demonstrated for the first time that mirogabalin relieves hypersensitivity when administered alone, but its anti-nociceptive effect is enhanced when it is coadministered with opioid drugs (morphine, buprenorphine, oxycodone) in a mouse model of neuropathic pain [20]. In 2025, clinical trials confirmed the efficacy of mirogabalin in cancer patients and its beneficial effects when combined with different opioid drugs. Furthermore, compared with pregabalin, mirogabalin with opioids contributes less to drowsiness and dizziness in patients [21].

To address this gap, the present study aimed to evaluate the analgesic potential of mirogabalin in a model of neuropathic pain and to compare its effects with antidepressants commonly used in the clinical management of neuropathic pain (amitriptyline and duloxetine) in mice following chronic constriction injury (CCI) of the sciatic nerve. Given their distinct mechanisms of action described above we hypothesized that their combined use may produce complementary antinociceptive effects and improve therapeutic outcomes.

## Materials and methods

### Animals

For the experimental procedures, we used male Swiss Albino mice (provided by Charles River, Göttingen, Germany; 9–11 weeks old, weighing 20–25 g). The mice were kept in groups of 8–10 per cage with free access to food and water. The following housing conditions were ensured: temperature (22±2°C), relative humidity (55±10%), and a 12-h light/12-h dark cycle. Animals were randomly allocated to experimental groups. Behavioral assessments were not performed under blinded conditions. Mice were acclimatized to the housing conditions for at least 5 days prior to the experiments. The number of animals was reduced to the essential minimum, consistent with the 3 R policy. All procedures were performed according to the recommendations of the National Institutes of Health (NIH) Guide for the Care and Use of Laboratory Animals and the International Association for the Study of Pain (IASP) and were approved by the Ethical Committee of the Maj Institute of Pharmacology, Polish Academy of Sciences (permission numbers: 192/2022; 142/2025). The manuscript has been revised to ensure compliance with the ARRIVE guidelines.

### Chronic constriction injury of the sciatic nerve

Chronic constriction injury (CCI) of the sciatic nerve has been performed to mimic neuropathic pain symptoms [22]. Mice that underwent surgical procedures were anesthetized by inhalation of isoflurane (induction, 4%; maintenance, 4%). In brief, after the skin was shaved and disinfected, an incision was created below the right hip bone. Afterward, the muscles were separated with tweezers, and the sciatic nerve was exposed. Next, the nerve was loosely tied with three knots (4/0 silk). The level of nerve tightening was dictated by the occurrence of a short contraction in the corresponding hind limb after the first knot was tied, and the subsequent knots were performed similarly. All the mice developed neuropathic pain symptoms, which manifested as tactile and thermal hypersensitivity.

### Pharmacological studies

#### Intraperitoneal drug administration

Intraperitoneal (*ip*) administration was performed using standard procedures that are commonly applied in rodent studies. Briefly, each mouse was gently restrained, and the injection was delivered into the lower abdominal quadrant to ensure intraperitoneal delivery while minimizing discomfort. Substances were administered using 0.45 × 12 mm needles, with doses adjusted to individual body weight. All drugs used were dissolved in water for injection, which served as the vehicle (V). Naive mice (N) served as controls for CCI-exposed animals.

#### Single intraperitoneal administration of mirogabalin, amitriptyline, and duloxetine to mice with chronic constriction injury-induced neuropathy

Single *ip* administrations of mirogabalin (mirogabalin; MedChemExpress, United States) at doses of 5, 15, 30 and 60 mg/kg, amitriptyline (amitriptyline hydrochloride; Merck, Darmstadt) at doses of 1, 5, and 10 mg/kg and duloxetine (duloxetine hydrochloride; MedChemExpress, United States) at doses of 10, 20, and 30 mg/kg were performed on Day 7 after CCI, when mechanical and thermal hypersensitivity had fully developed. The effects of selected drugs on the development of tactile hypersensitivity were measured using the von Frey test, whereas thermal hypersensitivity was measured using the cold plate test after 0.5, 1, 3, 6, and 24 hours.

#### Repeated intraperitoneal administration of mirogabalin, amitriptyline, and duloxetine to mice with chronic constriction injury-induced neuropathy

A pre-emptive treatment strategy was applied to evaluate the ability of mirogabalin to prevent the development of neuropathic pain after CCI, including potential modulation of the underlying pathophysiological processes, and was given twice daily based on the observations described above. The drugs were administered at the following doses selected based on studies of their single injections: mirogabalin (10 mg/kg), amitriptyline (5 mg/kg), and duloxetine (10 mg/kg). The animals were divided into two groups and received vehicle or mirogabalin as a preemptive treatment at 16 h and 1 h before surgery. On the day of CCI, the mice received vehicle or mirogabalin after the procedure, and for the next 6 days, they received drugs twice daily (morning and afternoon) according to the following scheme: in the morning, the first injection - mirogabalin or vehicle was performed, and after 1 h, injections of amitriptyline or duloxetine were conducted. This approach resulted in the formation of 7 experimental groups: 1) the naive group and the CCI-exposed group, which were treated with 2) vehicle + vehicle, 3) mirogabalin + vehicle, 4) vehicle + amitriptyline, 5) mirogabalin + amitriptyline, 6) vehicle + duloxetine, and 7) mirogabalin + duloxetine. In the afternoon, a single injection of mirogabalin or vehicle was administered. The behavioral tests were conducted 3 h after the morning administration of mirogabalin or vehicle and 2 h after the administration of amitriptyline, duloxetine, or vehicle, to capture peak effects, on the 2nd, 4th, and 6th days after CCI.

### Behavioral tests

#### von Frey test

The level of hypersensitivity to mechanical stimuli (mimicking human allodynia) was measured using calibrated nylon monofilaments (ranging from 0.6 to 6 g; Stoelting, USA) as described previously [23]. The mice were placed in plastic cages with a wire mesh floor before the experiment. After 5 min of adaptation, the filaments were applied to the midplantar surface of the ipsilateral (right) hind paw, in order of increasing pressure [g], until the stimulation elicited hind paw withdrawal (rapid movement, licking, shaking). In the case of naive mice, two paws were tested.

#### cold plate test

The level of hypersensitivity to thermal stimuli (mimicking human hyperalgesia) was measured with a cold plate/hot plate analgesia meter (Ugo Basile, Italy). The temperature of the plate surface was maintained at 2 °C. The animals were placed on a cold plate surface until the stimulation elicited a hind paw withdrawal (lift) as previously described [23]. The latency was registered. The cutoff latency, meaning the maximal time for the mouse to be kept on the plate surface, was 30 seconds. In every animal exposed to CCI, the injured foot was the first to react. In the case of naive mice, two paws were measured.

### Data analysis

**Fig. 1:**
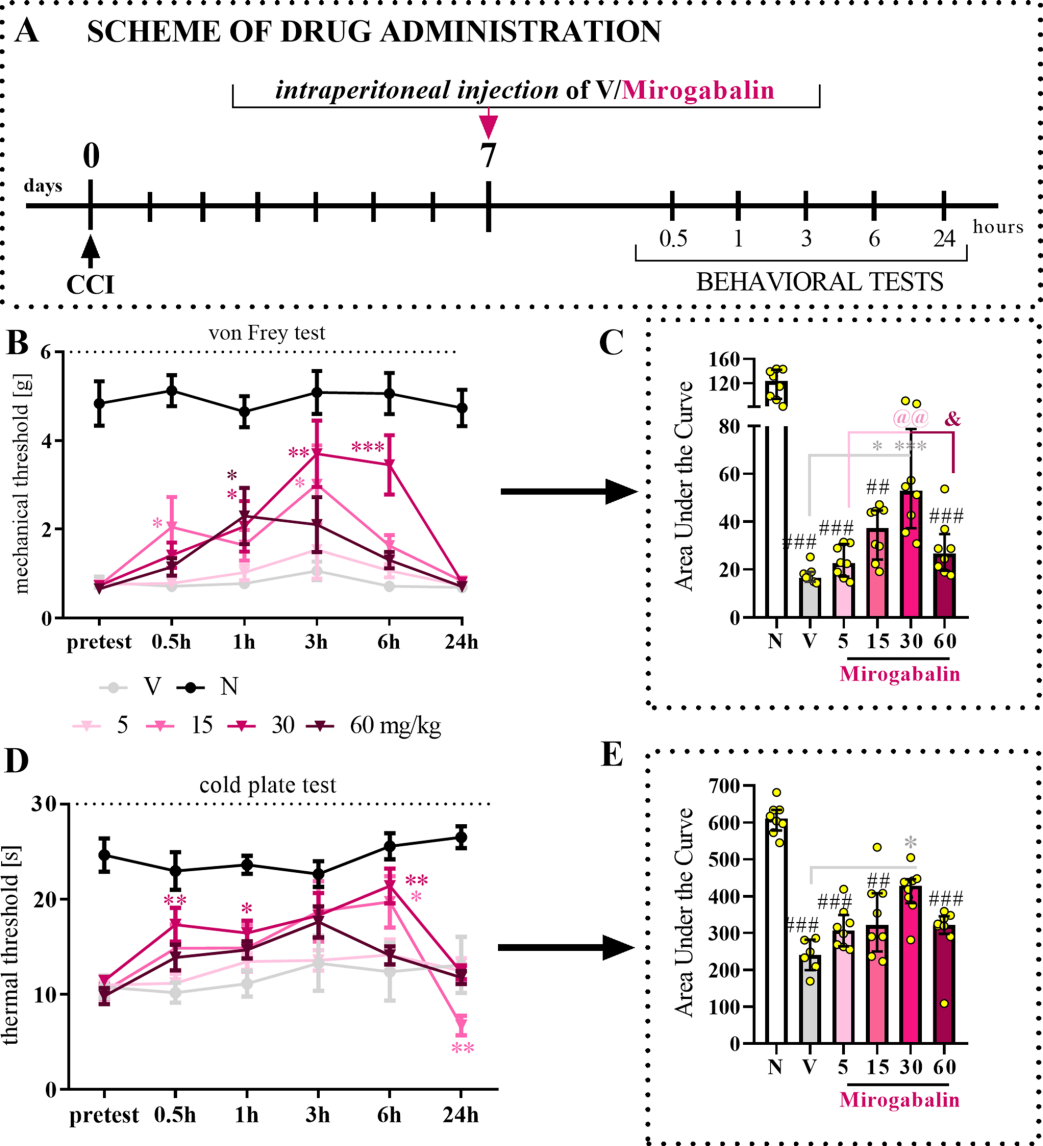
*Influence of single intraperitoneal mirogabalin administration on pain-related behavior* (**A**). The effects of mirogabalin injection (5, 15, 30, and 60 mg/kg) on tactile and thermal hypersensitivity were assessed at 0.5, 1, 3, 6, and 24 h after treatment on Day 7 after CCI in mice (**B**, **D**). Additionally, the data obtained from the von Frey (**B**) and cold plate (**D**) tests were analyzed as areas under the curve (**C**, **E**) to visualize overall changes between doses. The data of the dose-dependency study (**B**, **D**) are presented as the means ± SEMs, while the AUCs (**C**, **E**) are shown as the median + interquartile range (showing all individual points). The numbers of animals are as follows: *N* = 7–8; V = 6–7; 5 mg/kg = 7–8; 15 mg/kg = 8; 30 mg/kg = 8; and 60 mg/kg = 7–8. The intergroup differences of the study on dose-dependent mirogabalin efficacy were analyzed using one-way ANOVA with Bonferroni’s multiple comparisons test, while the AUCs were compared with the use of the nonparametric test (the Kruskal‒Wallis test with Dunn’s multiple comparisons). * *p* < 0.05, ** *p* < 0.01, and *** *p* < 0.001 indicate differences between V-treated CCI-exposed mice and substance-treated CCI-exposed mice; ## *p* < 0.01, ### *p* < 0.001 indicate differences compared with naive mice; @@ *p* < 0.01 indicates differences between animals treated with mirogabalin at a dose of 5 mg/kg; & *p* < 0.05 indicates differences between animals treated with mirogabalin at a dose of 30 mg/kg. The dotted lines represent cutoff values. Abbreviations: N, naive; V, vehicle, water for injection; CCI, chronic constriction injury of the sciatic nerve

**Fig. 2:**
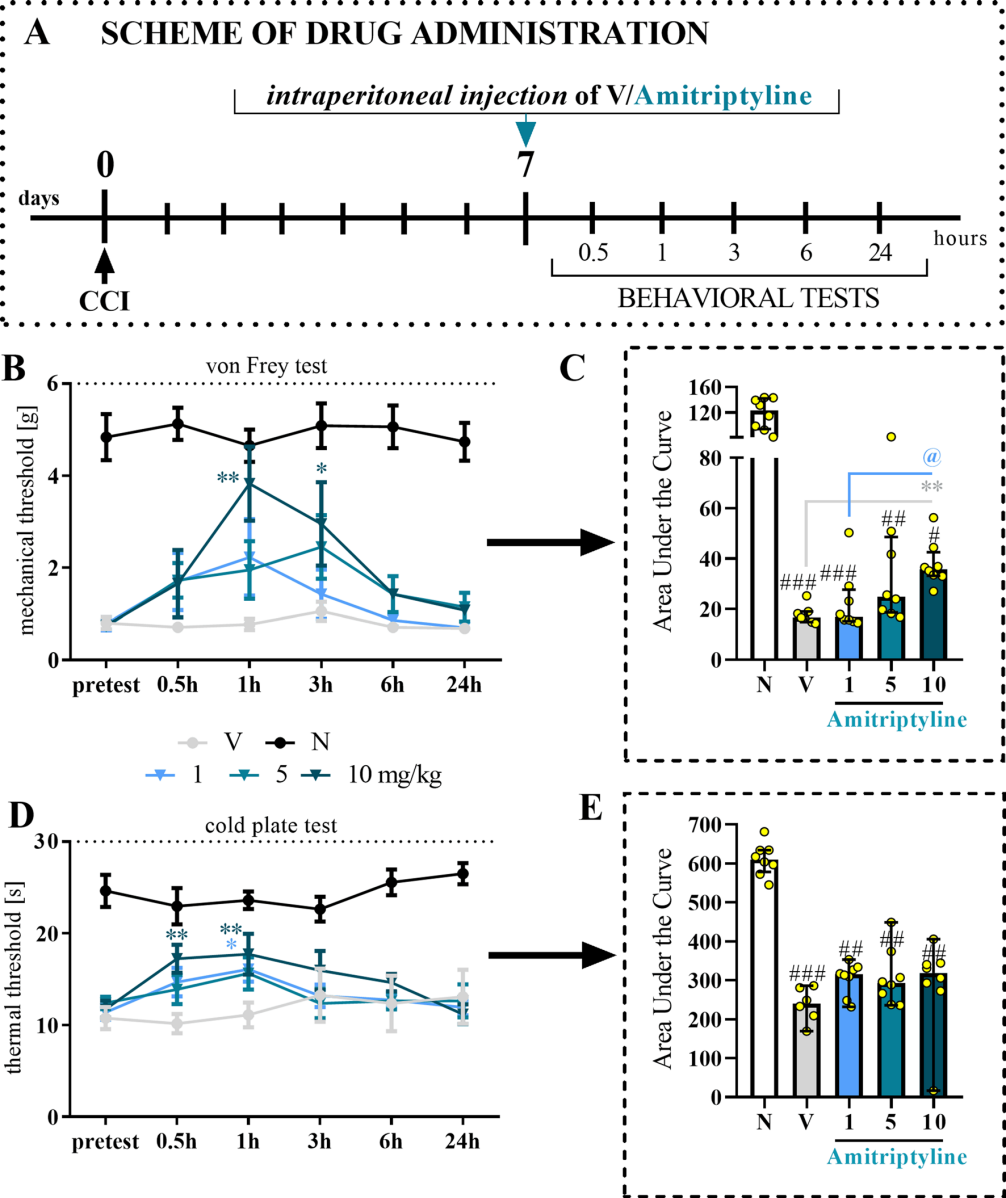
*Influence of single intraperitoneal amitriptyline administration on pain-related behavior in* (**A**). The effects of amitriptyline injections (1, 5, and 10 mg/kg) on tactile and thermal hypersensitivity were assessed at 0.5, 1, 3, 6, and 24 h after treatment on Day 7 after CCI in mice (**B**, **D**). Additionally, the data obtained from the von Frey (**B**) and cold plate (**D**) tests were analyzed as areas under the curve (**C**, **E**) to visualize overall changes between doses. The data of the dose-dependency study (**B**, **D**) are presented as the means ± SEMs, while the AUCs (**C**, **E**) are shown as the median + interquartile range (showing all individual points). The numbers of animals are as follows: *N* = 7–8; V = 6–7; 5 mg/kg = 7–8; 15 mg/kg = 8; 30 mg/kg = 8; and 60 mg/kg = 7–8. The intergroup differences of the study on dose-dependent amitriptyline efficacy were analyzed using one-way ANOVA with Bonferroni’s multiple comparisons test, while the AUCs were compared with the use of the nonparametric test (the Kruskal‒Wallis test with Dunn’s multiple comparisons).* *p* < 0.05, ** *p* < 0.01 indicate differences between V-treated CCI-exposed mice and substance-treated CCI-exposed mice; # *p* < 0.05, ## *p* < 0.01 and ### *p* < 0.001 indicate differences compared with naive mice; @ *p* < 0.05 indicates differences between animals treated with amitriptyline at a dose of 1 mg/kg. The dotted lines represent cutoff values. Abbreviations: N, naive; V, vehicle, water for injection; CCI, chronic constriction injury of the sciatic nerve

**Fig. 3:**
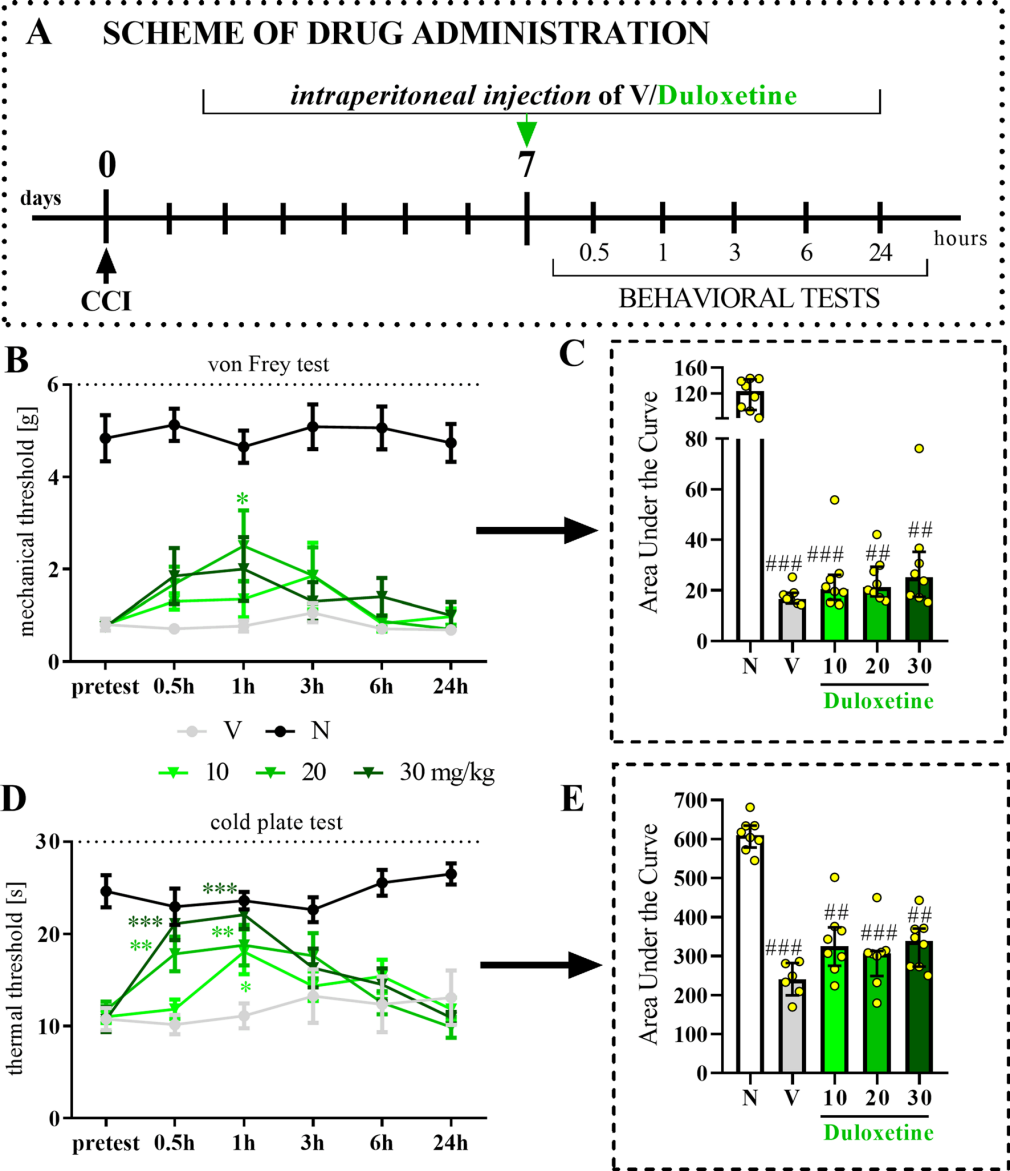
*Influence of single intraperitoneal duloxetine administration on pain-related behavior* (**A**). The effects of duloxetine injections (10, 20, and 30 mg/kg) on tactile and thermal hypersensitivity were assessed at 0.5, 1, 3, 6, and 24 h after treatment on Day 7 after CCI in mice (**B**, **D**). Additionally, the data obtained from the von Frey (**B**) and cold plate (**D**) tests were analyzed as areas under the curve (**C**, **E**) to visualize overall changes between doses. The data of the dose-dependency study (**B**, **D**) are presented as the means ± SEMs, while the AUCs (**C**, **E**) are shown as the median + interquartile range (showing all individual points). The numbers of animals are as follows: *N* = 7–8; V = 6–7; 5 mg/kg = 7–8; 15 mg/kg = 8; 30 mg/kg = 8; and 60 mg/kg = 7–8. The intergroup differences of the study on dose-dependent duloxetine efficacy were analyzed using one-way ANOVA with Bonferroni’s multiple comparisons test, while the AUCs were compared with the use of the nonparametric test (the Kruskal‒Wallis test with Dunn’s multiple comparisons). * *p* < 0.05, ** *p* < 0.01, and *** *p* < 0.001 indicate differences between V-treated CCI-exposed mice and substance-treated CCI-exposed mice; ## *p* < 0.01 and ### *p* < 0.001 indicate differences compared with naive mice. The dotted lines represent cutoff values. Abbreviations: N, naive; V, vehicle, water for injection; CCI, chronic constriction injury of the sciatic nerve

**Fig. 4:**
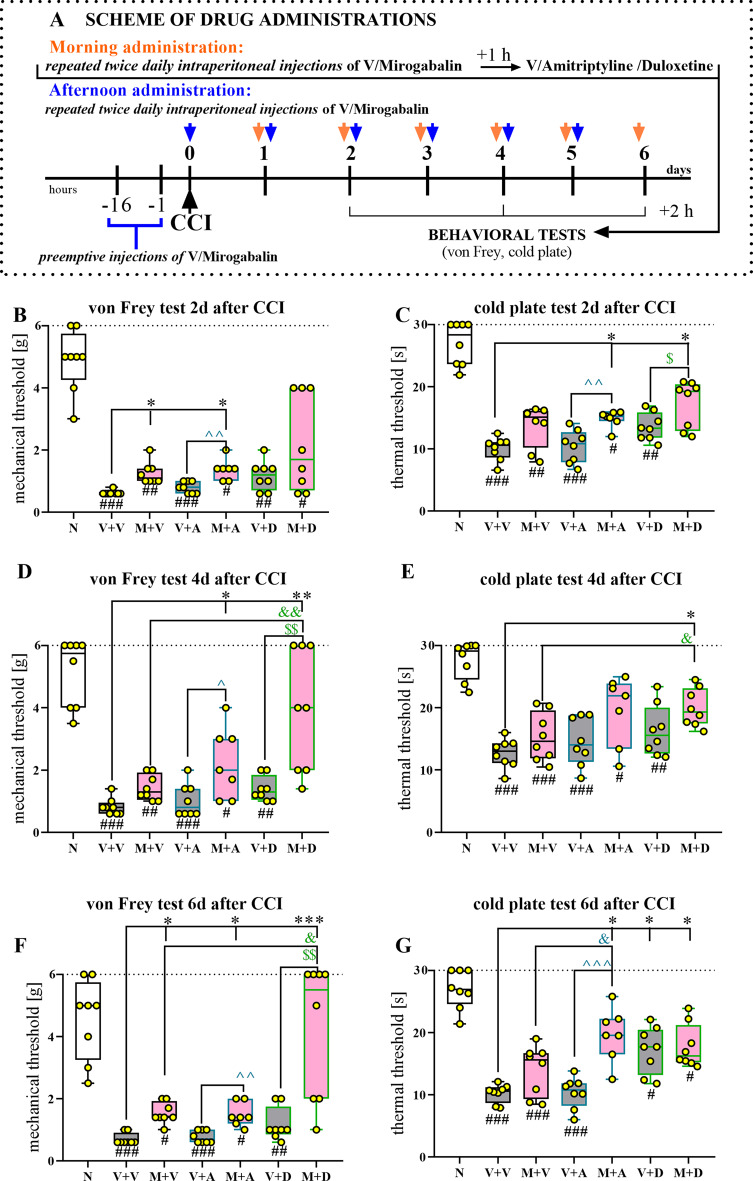
*Influence of chronic, twice-daily, combined intraperitoneal administration of mirogabalin with once-daily amitriptyline or duloxetine for 6 consecutive days on mechanical and thermal hypersensitivity, as measured by von Frey and cold plate tests, in neuropathic mice* (**A**). The day before CCI, the animals were divided into two groups: those receiving vehicle (V) and those receiving mirogabalin (10 mg/kg.; M) as a preemptive treatment. On the following days in the morning, the mice were administered V or M as the first injection, and after 1 h, they were injected with amitriptyline (5 mg/kg A), duloxetine (10 mg/kg. D), or vehicle (**V**). In the afternoon, the mice received V or M. The following seven experimental groups were used: N (*n* = 8), V+V (*n* = 8), **M**+**V** (*n* = 8), V+A (*n* = 8), **M**+**A** (*n* = 7), **V**+**D** (*n* = 8), and **M**+**D** (*n* = 8), which were measured with von Frey (**B**, **D**, **F**) and cold plate tests (**C**, **E**, **G**) on Days 2, 4 and 6 after CCI. The data are presented as the median + minimum and maximum values (showing all individual points). The results were analyzed using a nonparametric test (the Kruskal‒Wallis test with Dunn’s multiple comparisons). Differences between groups are presented as follows: #*p* < 0.05, ##*p* < 0.01, ###*p* < 0.001 indicate differences compared with naive mice; *p < 0.05, ** *p* < 0.01, *** *p* < 0.001 indicate differences between V-treated CCI-exposed mice and substance-treated CCI-exposed mice; ^*p* < 0.05, ^^*p* < 0.01, ^^^*p* < 0.001 indicate differences vs. **V**+**A** group; ^$^*p* < 0.05, ^$$^*p* < 0.01 indicate differences vs. V+D group, &*p* < 0.05, &&*p* < 0.01 indicate differences vs. **M**+**V** group. The dotted lines represent cutoff values. Abbreviations: **N**, naive; **V**, vehicle, water for injection; **M**, mirogabalin; **A**, amitriptyline; **D**, duloxetine; **CCI**, chronic constriction injury of the sciatic nerve

The Shapiro‒Wilk test was performed to evaluate the normality of the variable distributions (significance level α = 0.01). To evaluate the results from single administrations (mirogabalin, Fig. 1; amitriptyline, Fig. 2; or duloxetine, Fig. 3), one-way analysis of variance (ANOVA) was used, followed by Bonferroni post hoc correction of selected pairs, which were measured separately at each time point. Here, the behavioral data are presented as the means ± SEM in grams or seconds. The results shown in Figs. 1, 2, and 3 were additionally evaluated using two-way repeated measures ANOVA to detect time–drug interactions. The results of chronic administration of mirogabalin together with amitriptyline or duloxetine (Fig. 4) were evaluated using the Kruskal–Wallis test followed by the Dunn test. In that case, the data are presented as the median + minimum and maximum values. The area under the curve (AUC) results are also shown in the insert to visualize the efficacy of each dose (Figs. 1–3) as the median + interquartile range (showing all individual points), and the data were evaluated by the Kruskal–Wallis test followed by the Dunn test. The AUC was calculated via trapezoidal and Simpson’s rules, as described previously [24]. The total number of animals used in the present study was 166. All the statistical analyses and graphs were performed using Prism (GraphPad Prism 8.1.1, GraphPad Software, Inc., San Diego, CA, USA). Differences were considered significant when *p* < 0.05.

## Results

### Effects of a single intraperitoneal administration of mirogabalin on hypersensitivity to tactile and thermal stimuli on day 7 after CCI in mice

A single *ip* administration of mirogabalin was performed on Day 7 after CCI, and on that day, behavioral tests were conducted (Fig. 1A). According to the results of the von Frey test, mirogabalin reduced hypersensitivity to mechanical stimuli at doses of 15, 30, and 60 mg/kg. The most effective treatment was mirogabalin at a dosage of 30 mg/kg, which significantly diminished the tactile hypersensitivity starting from 1 h (F_5,41_ = 10.92; *p* < 0.0001); while the strongest effect of this dose was observed 3–6 h after administration (F_5,41_ = 5.228; *p* = 0.0008 and F_5,41_ = 20.81; *p* < 0.0001, respectively). A lower dose of mirogabalin (15 mg/kg) also resulted in antinociceptive effects at 0.5 and 3 h after the injection (F_5,41_ = 22.32; *p* < 0.0001 and F_5,41_ = 5.228; *p* = 0.0008, respectively), but not after 1 h. In turn, the highest dose of mirogabalin was effective only at one time point—after 1 h (F_5,41_ = 10.92; *p* < 0.0001) (Fig. 1B). Moreover, analysis of the AUC revealed that compared with vehicle treatment alone, the doses of 15 and 30 mg/kg mirogabalin significantly affected hypersensitivity to mechanical stimuli. What is more, the dose of 30 mg/kg was significantly more potent that the doses of 5 and 60 mg/kg mirogabalin (H = 34.53, N_1_ = 8, N_2_ = 7, N_3_ = 8, N_4_ = 8, N_5_ = 8, N_6_ = 8; *p* < 0.0001) (Fig. 1C). Two-way repeated measures ANOVA confirmed a significant interaction between the investigated treatment and the tested time points (F_25,246_ = 1.871; *p* = 0.0089).

Mirogabalin was also shown to reduce thermal hypersensitivity at two doses, 15 and 30 mg/kg, as observed in the cold plate test. The most effective treatment was again 30 mg/kg, which significantly diminished thermal hypersensitivity at 0.5, 1, and 6 h after administration. The strongest effect of the 30 mg/kg dose was observed at 0.5 h (F_5,41_ = 7.182; *p* < 0.0001) and 6 h (F_5,40_ = 6.210; *p* = 0.0002) after administration. A lower dose of mirogabalin (15 mg/kg) also resulted in antinociceptive effects, but only at 6 h after the injection (F_5,40_ = 6.210; *p* = 0.0002) (Fig. 1D). Similarly, analysis of the AUC revealed that compared with vehicle, only 30 mg/kg mirogabalin significantly affected hypersensitivity to thermal stimuli in mice subjected to CCI (H = 30.17, N_1_ = 8, N_2_ = 6, N_3_ = 8, N_4_ = 8, N_5_ = 8, N_6_ = 8; *p* < 0.0001) (Fig. 1E). Two-way repeated measures ANOVA confirmed a significant interaction between the investigated treatment and the tested time points (F_25,242_ = 2.032; *p* = 0.0035).

### Effects of a single intraperitoneal administration of amitriptyline on hypersensitivity to tactile and thermal stimuli on day 7 day after CCI in mice

A single *ip* administration of amitriptyline was performed on Day 7 after CCI, and on that same day, behavioral tests were performed (Fig. 2A). Amitriptyline had antinociceptive properties, resulting in diminished hypersensitivity to mechanical and thermal stimuli. According to the results of the von Frey test, the drug reduced the development of tactile hypersensitivity after CCI only at a dose of 10 mg/kg, 1 and 3 h after *ip* injection (F_4,34_ = 5.901; *p* = 0.0010; F_4,34_ = 6.407; *p* = 0.0006, respectively) (Fig. 2B). In the cold plate test, the effect of amitriptyline was observed between 0.5 h and 1 h. Amitriptyline was effective against thermal hypersensitivity at doses of 10 mg/kg after 0.5 and 1 h (F_4,34_ = 8.635; *p* < 0.0001; F_4,34_ = 7.665; *p* = 0.0002, respectively) and at a dose of 1 mg/kg after 1 h (F_4,34_ = 7.665; *p* = 0.0002) (Fig. 2D). Analysis of the AUC revealed that the dose of 10 mg/kg *ip* of amitriptyline had a significant effect only on mechanical hypersensitivity in mice subjected to CCI compared with groups treated with vehicle and the lower dose of 1 mg/kg (H = 27.23, N_1_ = 8, N_2_ = 7, N_3_ = 8, N_4_ = 8, N_5_ = 8; *p* < 0.0001) (Fig. 2C); however, AUC analysis of the data obtained in the cold plate test showed no differences between the doses (Fig. 2E). Two-way repeated measures ANOVA did not confirm a significant interaction between the investigated treatment and the tested time points in case of von Frey test and cold plate tests.

### Effects of a single intraperitoneal injection of duloxetine on hypersensitivity to tactile and thermal stimuli on day 7 after CCI in mice

A single *ip* administration of duloxetine was performed on Day 7 after CCI, and on that same day, behavioral tests were performed (Fig. 3A). Duloxetine had antinociceptive effects and diminished hypersensitivity to mechanical stimuli at a dose of 20 mg/kg 1 h after injection (F_4,34_ = 7.635; *p* = 0.0002) (Fig. 3B). Two-way repeated measures ANOVA did not confirm a significant interaction between the investigated treatment and the tested time points.

In turn, duloxetine diminished thermal hypersensitivity to stimuli at doses of 10, 20, and 30 mg/kg, as shown by the results of the cold plate test. The most effective dose of duloxetine was 30 mg/kg, which significantly prevented cold hypersensitivity after 0.5 and 1 h (F_4,34_ = 11.60; *p* < 0.0001; F_4,34_ = 6.743; *p* = 0.0004, respectively); similarly, the dose of 20 mg/kg was effective at the same time points (F_4,34_ = 11.60; *p* < 0.0001 after 0.5 h; F_4,34_ = 6.743; *p* = 0.0004 after 1 h). Duloxetine at the lowest dose used (10 mg/kg) also had antinociceptive effects 1 h after injection (F_4,34_ = 6.743; *p* = 0.0004) (Fig. 3D). Two-way repeated measures ANOVA confirmed a significant interaction between the investigated treatment and the tested time points (F_20,202_ = 2.803; *p* = 0.0001). Analysis of the AUC for the data obtained in both tests showed no differences between the doses (Fig. Fig. 3C, E).

### Effects of chronic, twice-daily intraperitoneal administration of mirogabalin and once-daily amitriptyline or duloxetine on hypersensitivity to tactile and thermal stimuli on Days 4 and 6 after CCI in mice

Repeated *i.p.* administrations of mirogabalin, amitriptyline, and duloxetine were performed until 6 days after CCI, and on Days 2, 4, and 6, behavioral tests were conducted (Fig. 4A). After 2 days of administration, mirogabalin alone (**M+V**) produced antinociceptive effects and reduced mechanical hypersensitivity in CCI-exposed mice compared with the vehicle-treated group (**V+V**) (H = 21.20; N_1_ = 8; N_2_ = 8; N_3_ = 8; *p* < 0.0001). According to the results of the von Frey test, combined administration of mirogabalin with amitriptyline (**M+A**) was also significantly more effective than treatment with vehicle (**V+V**) (H = 20.35, N_1_ = 8, N_2_ = 8, N_3_ = 7, *p* < 0.0001) and amitriptyline alone (**V+A**) (H = 12.25, N_1_ = 8, N_2_ = 8, N_3_ = 7, *p* = 0.0022) against tactile hypersensitivity. In turn, treatment with mirogabalin combined with duloxetine (**M+D**) was not more potent than treatment with duloxetine alone (**V+D**) and other treatments (Fig. 4B). The results of the cold plate test on the Day 2 of chronic injections were slightly different because treatment with mirogabalin (**M+V**) did not diminish thermal hypersensitivity, which was comparable to that in vehicle-treated mice. Combined administration of mirogabalin with amitriptyline (**M+A**) was significantly more effective against thermal hypersensitivity than amitriptyline alone (**V+A**) (H = 9.354, N_1_ = 8, N_2_ = 8, N_3_ = 7, *p* = 0.0093) and vehicle (**V+V**) (H = 19.35, N_1_ = 8, N_2_ = 8, N_3_ = 7, *p* < 0.0001). The difference in thermal hypersensitivity was visible in the effectiveness of duloxetine and mirogabalin treatment (**M+D**), compared with duloxetine (**V+D**) (H = 4.297, N_1_ = 8, N_2_ = 8, N_3_ = 8, *p* = 0.1167) and vehicle-treated (**V+V**) mice (H = 20.26, N_1_ = 8, N_2_ = 8, N_3_ = 8, *p* < 0.0001) (Fig. 4C).

After the next two days of administrations, on Day 4 after CCI, mirogabalin alone (**M+V**) did not affect hypersensitivity to mechanical stimuli relative to the vehicle group (**V+V**). In contrast, the combined treatment with mirogabalin and amitriptyline (**M+A**) was significantly more effective in reducing tactile hypersensitivity than amitriptyline alone (**V+A**) (H = 6.229, N_1_ = 8, N_2_ = 8, N_3_ = 7, *p* = 0.0444) or vehicle treatment (**V+V**) (H = 18.22, N_1_ = 8, N_2_ = 8, N_3_ = 7, *p* < 0.0001), as demonstrated in the von Frey test. Treatment with mirogabalin and duloxetine (**M+D**) was substantially more potent than single duloxetine treatment (**V+D**) (H = 11.08, N_1_ = 8, N_2_ = 8, N_3_ = 8, *p* = 0.0039); moreover, it was more effective than treatment with mirogabalin alone (**M+V**) (H = 11.08, N_1_ = 8, N_2_ = 8, N_3_ = 8, *p* = 0.0039) (H = 16.18, N_1_ = 8, N_2_ = 8, N_3_ = 8, *p* = 0.0008) (Fig. 4D). The results of the cold plate test, which was performed on Day 4 of chronic administration, differed from those of the earlier time point because only the treatment composed of mirogabalin and duloxetine (**M+D**) diminished thermal hypersensitivity vs. the groups that received mirogabalin alone (**M+V**) (H = 5.886, N_1_ = 8, N_2_ = 8, N_3_ = 8, *p* = 0.0527) and vehicle (**V+V**) (H = 19.57, N_1_ = 8, N_2_ = 8, N_3_ = 8, *p* < 0.0001) (Fig. 4E).

After the other two days of administration, on Day 6 after CCI, the situation differed between the two tests. According to the results of the von Frey test, compared with vehicle (**V+V**), mirogabalin alone (**M+V**) diminished hypersensitivity to mechanical stimuli (H = 20.66; N_1_ = 8; N_2_ = 8; N_3_ = 8; *p* < 0.0001). Mirogabalin, together with amitriptyline (**M+A**), was more effective against mechanical hypersensitivity than amitriptyline alone (**V+A**) (H = 14.29, N_1_ = 8, N_2_ = 8, N_3_ = 7, *p* = 0.0008) and vehicle (**V+V**) (H = 19.72, N_1_ = 8, N_2_ = 8, N_3_ = 7, *p* < 0.0001). Treatment with mirogabalin with duloxetine **(M+D**) was substantially more potent than duloxetine treatment alone (**V+D**) (H = 10.82, N_1_ = 8, N_2_ = 8, N_3_ = 8, *p* = 0.0045); moreover, it was more than treatment with mirogabalin alone (**M+V**) (H = 10.82, N_1_ = 8, N_2_ = 8, N_3_ = 8, *p* = 0.0045) and vehicle (**V+V**) (H = 15.45, N_1_ = 8, N_2_ = 8, N_3_ = 8, *p* = 0.0004) (Fig. 4F). The cold plate test performed on Day 6 of chronic administrations revealed that mirogabalin administered together with amitriptyline (**M+A**) was more effective in comparison with several treatments: mirogabalin alone (**M+V**) (H = 11.93, N_1_ = 8, N_2_ = 8, N_3_ = 7, *p* = 0.0026), amitriptyline alone (**V+A**) (H = 11.93, N_1_ = 8, N_2_ = 8, N_3_ = 7, *p* = 0.0026) and vehicle (**V+V**) (H = 18.39, N_1_ = 8, N_2_ = 8, N_3_ = 7, *p* < 0.0001). Compared with the vehicle-treated group, treatment with mirogabalin and duloxetine **(M+D**) (H = 19.89, N_1_ = 8, N_2_ = 8, N_3_ = 8, *p* < 0.0001) and duloxetine alone (**V+D**) (H = 19.89, N_1_ = 8, N_2_ = 8, N_3_ = 8, *p* < 0.0001) provided a more potent effect against thermal hypersensitivity (Fig. 4G).

## Discussion

Results of the present study suggest that a single *ip* administration of mirogabalin, amitriptyline, and duloxetine reduces CCI-evoked tactile and thermal hypersensitivity. Notably, after a single *ip* administration of mirogabalin, the anti-nociceptive effect lasted up to 6 hours, persisting longer than the effect of antidepressants. Next, we selected low doses for drug coadministration to test whether such a combination would enhance the pain-relieving properties of these drugs in patients with neuropathic pain. Our results imply that repeated administration of low doses of mirogabalin and antidepressants exerted a weak anti-nociceptive effect, if any. However, we observed enhanced anti-nociceptive efficacy in the group that received the combination of mirogabalin with duloxetine, based on the von Frey test, and in the group receiving mirogabalin with amitriptyline, based on the cold plate test. This finding was an important finding because duloxetine and amitriptyline are valuable drugs in clinical practice, and combination regimens with mirogabalin might allow for the use of lower doses and protect patients from drug-induced adverse events. The observed enhanced anti-nociceptive effect after combined administration of mirogabalin together with amitriptyline or duloxetine may be caused by operating at different levels of pain processing. Mirogabalin causes reduced presynaptic calcium ion influx, reducing the release of excitatory neurotransmitters in nociceptive pathways. Amitriptyline and duloxetine, in turn, increase the synaptic availability of serotonin and norepinephrine by modulating descending inhibitory monoaminergic pathways. Simultaneous modulation of complementary mechanisms operating at different levels of pain processing may therefore contribute to the enhanced anti-nociceptive response observed following combined treatment, which may be particularly important in neuropathic pain conditions.

Mirogabalin besylate ([(1 R,5S,6S)-6-(aminomethyl)-3-ethylbicyclo[3.2.0]hept-3-en-6-yl] acetic acid) was developed by Daiichi Sankyo for the treatment of pain; the name of the drug is Tarliage, and it is already available on the market in some countries, currently in the dosage range of 2.5–15 mg. Mirogabalin binds selectively and with a high affinity to the α2δ-1/-2 subunits of VGCCs—its dissociation constant (Kd) is 13.5 nmol/l for the α2δ-1 subunit and 22.7 nmol/l for the α2δ-2 subunit [14]. Compared with pregabalin (Kd = 62.5 nmol/L), mirogabalin exhibits substantially higher affinity for α2δ-1 [14]. Moreover, mirogabalin binds to the subunits of VGCCs for a longer time—its dissociation half-life from the α2δ-1 and α2δ-2 subunits is 11.1 and 2.4 hours, respectively [14]. This slower dissociation rate for α2δ-1 than for α2δ-2 is considered a key factor underlying the enhanced analgesic efficacy and favorable safety profile of mirogabalin [25, 26]. On the basis of the literature, it seems that channels located on neuronal cells are responsible for the rapid anti-nociceptive effect of mirogabalin observed after a single dose [15, 27, 28]. These findings are supported by in vitro data showing that mirogabalin inhibits neuronal VGCC currents at a concentration of 50 μM, whereas pregabalin inhibits them at a concentration of 200 μM [28]. These findings correlate well with the anti-nociceptive properties reported in our previous studies, in which the ED_50_ for mirogabalin is lower than that for pregabalin [29]. Therefore, mirogabalin may be more effective than pregabalin in patients with neuropathic pain, and similar to pregabalin, may reduce the activation and damage of neurons [30]. However, due to the absence of a direct comparison with pregabalin (methodological limitations of the present study), these findings should be interpreted with caution. Notably, in nociplastic pain arising from dysregulation of descending pain-modulatory pathways [31], administration of mirogabalin and pregabalin reduced bilateral hind-limb hypersensitivity in the von Frey test, which is thought to result from their binding to α2δ-1 subunits in nervous system regions involved in central pain processing [32]. Structural and functional changes in central nervous system structures also occur during the development of neuropathic pain, and various neurotransmitter systems are involved in pain pathophysiology [33]. In our studies, we have already shown that repeated administration of mirogabalin in mice exposed to CCI reduces the levels of pp38/p38, the MAP kinase that is known to be crucial for pain development [34–36]. For example, pharmacological studies have shown that substances that can inhibit p38, such as minocycline, SB203580, CNI-1493, and FR167653, prevent and/or diminish neuropathic pain symptoms [23, 34, 37–45]. Furthermore, p38 is mostly activated in microglia at the spinal cord level during neuropathy [34, 36, 43, 44, 46]. Moreover, highly activated microglia are known to be the source of many pronociceptive factors; thus, mirogabalin silences neuroimmune changes, most likely by silencing neuronal cells [47]. All available studies provide evidence that mirogabalin has a unique and broad spectrum of action. Moreover, it is absorbed very rapidly after administration, with a mean time to reach maximum plasma concentration (Tmax) of 0.5–1.5 hours [48]. Furthermore, the mean half-life of mirogabalin reported in various clinical studies is 2–3 [13], 3.5 [49], or 3–5 [50] hours. These features could explain why, in our study, mirogabalin demonstrated anti-nociceptive efficacy for up to 6 hours; moreover, for this reason, mirogabalin was administered repeatedly twice daily in our experiments. The obtained behavioral results were consistent with those of previous studies showing that mirogabalin has potent anti-nociceptive effects after oral [51] and intraperitoneal [20, 29] administration in rats/mice exposed to CCI. Moreover, mirogabalin has already been shown to be effective at reducing pain in animal models of spinal cord injury [14], posttraumatic trigeminal neuropathy [52], and formalin-induced inflammation [53]. Furthermore, in 2019, mirogabalin was demonstrated to be a potent and long-lasting anti-nociceptive in Sluka and intermittent cold stress models of fibromyalgia [54], where it was administered orally at doses ranging from 1 to 10 mg/kg; depending on the dose, it relieved pain for up to 8 hours, as measured by the von Frey test [54]. In addition, in 2021, another paper was published, providing evidence that oral administration of mirogabalin (3–10 mg/kg) improved cognitive impairment in a rat Sluka model [55]. Therefore, mirogabalin may alleviate not only pain but also cognitive impairments in patients with fibromyalgia [54, 55]. Importantly, the results of a recently published review highlight the benefits of using two drugs, mirogabalin and duloxetine, in the treatment of fibromyalgia patients [11]. Recently, the first clinical data have emerged suggesting improved effects of the combined use of mirogabalin and duloxetine, for example, in patients with chemotherapy-induced peripheral neuropathy [56, 57], but experimental studies in models of neuropathy of different etiology are still lacking. However, several lines of evidence support the efficacy of other gabapentinoids in treating neuropathic pain of different etiologies [5, 6, 16–19, 58–60]. We would like to emphasize that mirogabalin was approved in Japan in 2019 for the treatment of pain in patients with diabetic neuropathy and postherpetic neuralgia [8]. Subsequently, in 2021, Japanese scientists published study results showing that switching from pregabalin to mirogabalin is well tolerated and effective in the treatment of neuropathic pain using stepwise titration [9]. The latest clinical studies, published in 2022, provide evidence that mirogabalin is very effective for relieving pain after spinal cord injury [10] and in 2024 in patients with fibromyalgia [11]. To date, mirogabalin is approved for the general indication of neuropathic pain in Japan, peripheral neuropathic pain in South Korea, and diabetic neuropathy/postherpetic neuralgia in China [12]. In Asian patients, mirogabalin administered at doses of up to 15 mg twice daily for 7 days is safe and well tolerated, and the reported side effects include drowsiness, headache, and dizziness [13]. Our experimental results revealed that mirogabalin at a dose of 10 mg/kg administered twice daily for 7 days diminished neuropathic pain with no visible side effects.

In 2025, mirogabalin was shown for the first time to be successfully used in the clinic to treat neuropathic pain in cancer patients [21]. It has been shown that compared with pregabalin, mirogabalin leads to a lower incidence of drowsiness and dizziness in cancer patients receiving strong opioids [21]. This finding is of particular interest in the context of our previous study demonstrating that mirogabalin could improve the anti-nociceptive effect induced by three opioids, morphine, buprenorphine, oxycodone, and ketamine, in a mouse model of neuropathic pain [20]. In our current work, we showed for the first time that mirogabalin could also improve the effectiveness of duloxetine and amitriptyline in the same model. Both of these antidepressants were administered in our studies at low-efficacy doses to test whether their combined administration with mirogabalin could contribute to a better anti-nociceptive effect in neuropathy. We selected two drugs from different groups of antidepressants, which are often used in clinical practice. Amitriptyline has been used for a long time in the clinic, and it provides the best anti-nociceptive results among TCAs [61], as it increases the concentrations of noradrenaline and serotonin in the synaptic cleft [3]. Increased levels of these neurotransmitters are caused by inhibition of their reuptake, which results from blocking specific transporters located in the presynaptic membrane [3, 62, 63]. Because noradrenaline and serotonin, as well as their receptors, are involved in the regulation of nociception at different levels of the nervous system, amitriptyline appears to enhance endogenous pain control by increasing the activity of the descending antinociceptive spinal tract [64], leading to an increased pain threshold. Moreover, amitriptyline is a tertiary amine with strong binding affinities for alpha-adrenergic, histamine type 1, and muscarinic type 1 receptors [65]. Amitriptyline is used to relieve neuropathic pain, often in combination with other medications, including gabapentinoids [16, 18, 19]. Our results indicated that the anti-nociceptive effect was superior in the group receiving mirogabalin combined with amitriptyline based on both the von Frey test and the cold plate test, with the best result achieved in the cold plate test on Day 6. Taken together, these results suggest that the combined use of low-dose mirogabalin with amitriptyline may provide therapeutic benefits in clinical practice, which requires further investigation.

Duloxetine is an antidepressant belonging to the SNRIs and is currently used for the treatment of chronic pain, especially in diabetic neuropathy [66], fibromyalgia [67, 68], osteoarthritis [69], among others [3, 70–73]. Duloxetine effectively relieves neuropathic pain, often in combination with pregabalin [17, 18] or gabapentin [19]. It increases noradrenaline concentrations in the synaptic cleft by inhibiting noradrenaline transporters in the CNS [7]. Spinal noradrenaline, increased by duloxetine, suppresses pain signals by substituting for the descending noradrenergic pain inhibitory system [74–76]. It has been shown that *ip* duloxetine increases spinal noradrenaline levels to produce an anti-nociceptive effect even when neuropathic pain is well established [77]. Enhancement of noradrenergic transmission appears to be crucial, although the serotonergic system also contributes, primarily *via* modulation of noradrenergic pathways [78, 79]. Recently, the combination of mirogabalin and duloxetine has been shown to reduce chemotherapy-induced peripheral neuropathic pain in patients with advanced lung cancer. These findings call for further investigations and consideration for their integration into clinical practice for the management of neuropathy caused by the use of anticancer agents [56]. A systematic review published in 2023 revealed that out of 25 antidepressants assessed across 176 studies with 28,664 patients, duloxetine was the only substance with confirmed effectiveness in the treatment of neuropathic pain, which is why it has the greatest degree of trust [61]. It probably has a moderate effect on improving physical function and reducing pain. For every 1000 patients taking a standard dose of duloxetine, 435 experienced 50% pain relief, which is certainly significant compared with 287 who experienced the same pain relief when taking a placebo. Notably, according to a meta-analysis, a standard dosage of duloxetine (60 mg per day) is effective for patients [61]. To potentiate the favorable influence of the drug on neuropathic patients, it is necessary to determine its better utility in combination with drugs with different mechanisms of action. The results obtained in our study indicated that after nerve injury in the von Frey test, duloxetine administered alone to mice at a dosage of 10 mg/kg had very little, if any, anti-nociceptive effect. Importantly, from Day 4 onward, the anti-nociception after the combined administration of mirogabalin and duloxetine was very good and was the most significant on Day 6. The results of the cold plate test were also promising; repeated administration of duloxetine in combination with mirogabalin initially provided better pain relief, but the effects on Day 6 were also very good; however, the results were comparable to those of duloxetine alone. In our opinion, these findings are critically important information for clinicians; the use of low doses of mirogabalin and duloxetine can provide significant therapeutic benefits and reduce the risk of drug-induced adverse effects.

## Summary

Mirogabalin is a novel gabapentinoid drug, which demonstrated good anti-nociceptive effects in our behavioral studies and has been previously shown to be superior to pregabalin [29] and to exert comparable effects compared with currently used first-line medications (amitriptyline and duloxetine). The absence of a direct comparison with pregabalin in our study restricts the strength of conclusions regarding comparative efficacy. However, the pharmacological effects of mirogabalin lasted longer than those of the other tested antidepressants, persisting up to 6 hours. Furthermore, considering the current results were obtained in a mouse model, we hypothesize that the combined administration of mirogabalin with amitriptyline or duloxetine may play a significant role in the planning of future neuropathic pain therapies. Given that mirogabalin is already used clinically in several countries for the treatment of neuropathic pain of various etiologies [8–13], we believe that it should be approved in other countries as well. Overall, these promising results are important for the development of new, more effective polytherapy interventions for neuropathic pain; however, further clinical trials are necessary.

## Data Availability

The data presented in this study are available upon request from the corresponding author.
